# Knowledge and Attitude of General Dentists, Senior Dental Students, and Orthodontic Residents Toward Obstructive Sleep Apnea

**DOI:** 10.1002/cre2.931

**Published:** 2024-09-18

**Authors:** Mina Shekarian, Masood Feizbakhsh, Mehdi Rafie

**Affiliations:** ^1^ School of Dentistry, Isfahan (Khorasgan) Branch Islamic Azad University Isfahan Iran; ^2^ Department of Orthodontics, Dental School, Isfahan (Khorasgan) Branch Islamic Azad University Isfahan Iran

**Keywords:** attitude, knowledge, obstructive sleep apnea

## Abstract

**Objective:**

The main objective of this study was to compare the knowledge and attitude of general dentists, senior dental students, and orthodontic residents toward obstructive sleep apnea (OSA).

**Materials and Methods:**

A questionnaire was designed and administered among 73 senior dental students, 84 general dentists, and 50 orthodontic residents. The questionnaire asked for demographic information of the participants and assessed their knowledge and attitude toward OSA. The validity and reliability of the questionnaire were confirmed by experts. Data were analyzed by the Kruskal−Wallis and Bonferroni tests using SPSS (*α *= 0.05).

**Results:**

The mean knowledge scores of general dentists and senior dental students were significantly lower than those of orthodontic residents (*p* < 0.001). However, there was no significant difference in the knowledge score of senior dental students and general dentists (*p* = 1). The mean knowledge score was significantly higher in dentists with 1–3 years of professional clinical experience (*p* = 0.02). The knowledge score was the highest in dentists working in private clinics followed by private offices and public clinics. The mean attitude score was the highest in orthodontic residents followed by general dentists and dental students. There was no significant difference in attitude based on the attended university, age, or time of graduation.

**Conclusion:**

According to the results, general dentists had insufficient knowledge about OSA, which could result in under‐diagnosis or misdiagnosis of patients with OSA. This finding highlights the need for further education and training for dentists to properly identify and refer OSA patients to orthodontists or sleep specialists.

## 1 Introduction

Obstructive sleep apnea (OSA) is the most common sleep disorder characterized by regular and repetitive, partial or complete upper airway obstruction (Amra et al. 2014; Foresman 2000), which results in oxygen depletion (Friedman, Landsberg, and Ascher‐Landsberg 2001).

A complete medical history, clinical examination, and a lateral cephalogram and/or cone beam computed tomography (CBCT) are often required for diagnosis of OSA (Friedman and Jacobowitz 2018; ki Beom et al. 2021). A comprehensive medical history that includes the presence/absence of symptoms, such as snoring, apnea, morning headaches, trouble in concentration, and daytime sleepiness can aid in the diagnosis of OSA (Kim and Kim 2020a).

Several studies have highlighted the significant role of dentists in the identification and management of patients with OSA (Smith and Smith 2017; Quan and Schmidt‐Nowara 2017; Luzzi et al. 2019; Padmanabhan, Kavitha, and Hegde 2010; Leibovitz et al. 2017). Dentists can be the first line of diagnosis of OSA by examining the size of the uvula, position, and size of the tongue, and soft palate length during routine clinical examinations, especially in patients who have regular check‐ups every 6 months (Kripke et al. 1997). Additionally, using a simple questionnaire, such as the Epworth Sleep Scale, BANG, STOP‐BANG, ARES, Berlin, and Pittsburgh Sleep Quality Index can be beneficial for the detection of OSA (Kim and Kim 2020a; Jinmei et al. 2014; Chervin et al. 2000).

It has been shown that untreated OSA can lead to serious health consequences, such as hypertension, diabetes mellitus, cardiovascular diseases, stroke, decreased quality of life, and even mortality (Friedman and Jacobowitz 2018; Knauert et al. 2015; Young et al. 1997; Chugh and Dinges 2002; Jenkinson, Stradling, and Petersen 1997). Knowledge in this regard is crucial for dental clinicians, as most complications of OSA can be prevented if it is diagnosed and managed correctly at an appropriate time. Moreover, dentists can play a vital role in the diagnosis and treatment of OSA through surgical and nonsurgical approaches or referral of patients, given that they receive proper training in this regard. The nonsurgical approaches include the use of oral appliances, while surgical approaches may include procedures, such as adenotonsillectomy, palatal surgery, hypopharyngeal surgery, and maxillomandibular advancement. Nonetheless, dentists appear to be underutilized in the management of OSA.

The prevalence of OSA in the Iranian population is as high as 44%, which is the highest in Asia (Sarokhani et al. 2019); therefore, early diagnosis of OSA is pivotal. There are numerous articles discussing the diagnosis and treatment of OSA in Iran; however, there is currently no specific guideline available in this area. General dentists in Iran do not receive any training regarding the diagnosis and treatment of OSA, unlike orthodontic residents who receive some education and training in this regard. The educational dental curriculum for general dentists in Iran does not include any learning objectives for OSA screening and management (The Ministry of Health and Medical Education 2012). Additionally, there is a lack of collaboration between medical and dental professionals when it comes to the diagnosis and treatment of OSA. To the best of the authors' knowledge, only one study is available on the knowledge and attitude of Iranian dentists toward OSA (Shafiei et al. 2020). Thus, the purpose of this study was to assess and compare the knowledge level and attitude of general dentists, senior dental students, and orthodontic residents toward OSA in Iran.

## 2 Materials and Methods

This cross‐sectional study was conducted from January 2021 to December 2023. The study population included 207 participants consisting of 84 general dentists, 73 senior dental students, and 50 orthodontic residents who were selected by simple random sampling. The total sample size was calculated assuming *α* = 0.05 and study power of 90%.

Of 43 dental schools in Iran, 9 were randomly selected. One researcher (M.S.H.) approached the Education and Training Faculty of the selected dental schools to receive the list of all attending dental students and orthodontic residents and their contact information. Also, the list of all practicing general dentists in the same cities was obtained from the medical council of the respective cities (Tehran, Isfahan, Shiraz, Yazd. Tabriz, Shahrekord, and Kashan). The participants were subsequently selected by cluster sampling from the lists and were provided with the online questionnaire. The selected individuals were provided with a consent form before receiving the questionnaire. The participants who agreed to participate were provided with the questionnaire.

The participants were briefed about the study and ensured the anonymity and confidentiality of their information. Written informed consent was obtained from the participants as well. They were also informed that participation in the study was voluntary, and they were free to quit at any time.

This study protocol was approved by the ethics committee of the Islamic Azad University of Isfahan (https://ethics.research.ac.ir/IR.IAU.KHUISF.REC.1400.264 code).

### 2.1 Questionnaire Development

A pre‐designed English questionnaire was selected from a literature review (Nguyen 2023). The experts translated it into Persian and confirmed its accuracy by the back‐translation method. The content validity, face validity, and reliability of the questionnaire and each question were evaluated by seven orthodontists and three pedodontists. The content validity of the questionnaire was assessed by calculation of the content validity ratio (CVR) and content validity index (CVI). To calculate the CVR, the experts rated each question as necessary (score 3), beneficial but not necessary (score 2), and not necessary (score 1). The CVI measured three indicators of relevance (CVI_R_) of the question to the topic, clarity (CVI_C_), and simplicity (CVI_S_).

According to the CVR formula, questions with a score < 0.62 were eliminated. CVI was also measured based on relevance, simplicity, and clarity. Only questions with a CVI higher than 0.79 were used in the final questionnaire (the CVI and CVR scores for each question are presented in Table 1).

**Table 1:** CVI and CVR scores for each question.

Question number	1	2	3	4	5	6	7	8	9	10	11
CVR	100%	60%	80%	60%	80%	80%	100%	100%	80%	80%	100%
CVI_R_	100%	100%	100%	70%	70%	80%	80%	90%	90%	90%	90%
CVI_S_	100%	100%	100%	100%	100%	100%	100%	100%	100%	100%	100%
CVI_C_	100%	100%	100%	80%	80%	100%	100%	100%	100%	100%	100%

Question number	12	13	14	15	16	17	18	19	20	21	22
CVR	80%	100%	100%	80%	80%	100%	100%	100%	80%	100%	100%
CVI_R_	90%	90%	90%	90%	70%	100%	100%	100%	80%	100%	100%
CVI_S_	100%	90%	100%	100%	100%	100%	100%	100%	100%	100%	100%
CVI_C_	90%	90%	100%	100%	100%	100%	100%	100%	100%	100%	100%

Question number	23	24	25	26	27	28	29	30	31	32	33
CVR	100%	80%	80%	100%	100%	80%	80%	80%	80%	100%	80%
CVI_R_	100%	80%	80%	100%	100%	80%	80%	80%	80%	100%	80%
CVI_S_	100%	100%	100%	100%	100%	100%	100%	100%	100%	100%	100%
CVI_C_	90%	100%	100%	100%	100%	100%	100%	100%	100%	100%	90%

It should be mentioned that 4 questions were omitted from a total of 33 primary questions due to low CVI or CVR scores (according to Table 1, questions #2, 4, 5, and 16 were omitted).

The test–retest reliability and Cronbach's *α* methods were used to evaluate the reliability of the knowledge domain and attitude domain of the questionnaire, respectively. The coefficients for the knowledge and attitude domains were calculated to be 0.987 and 0.715, respectively.

This final questionnaire had three main domains: demographic information, knowledge, and attitude. The demographic information domain included the age, sex, type of university, time passed since graduation, location of professional practice, and clinical professional experience. The first 23 questions belonged to the knowledge domain, which covered the definition, epidemiology, symptoms, diagnosis, and treatment of OSA. The next six questions belonged to the attitude domain, which discussed the role of dentists in the prevention, identification, and management of OSA.

The final questionnaire, presented in the supplementary file, was pilot‐tested on 10 participants. They did not offer any suggestions to change the questions. Thus, the researchers administered both printed and online questionnaires among the target groups. It took 8–10 min to complete the questionnaire.

### 2.2 Scoring of the Questionnaire

Each correct answer of the questionnaire was given a score of 1, while incorrect answers were scored 0. Questionnaires with unanswered questions were excluded. The knowledge score could range from 0 to 23 based on the knowledge level of the participants. Scores 0–6 indicated poor knowledge, scores 7–17 indicated moderate knowledge, and scores 18–23 indicated a high knowledge level of the participants.

The attitude score could range from 6 to 30. Scores 6–11 indicated a negative attitude while scores 25–30 indicated a positive attitude. Scores 12–24 indicated a neutral attitude.

Data were analyzed descriptively and inferentially using SPSS version 26.0. If the necessary assumptions were met at the inferential level, one‐way ANOVA was used for the comparisons. Otherwise, the Kruskal−Wallis test was applied. At the descriptive level, the mean and standard deviation values were reported to compare the distribution of the samples. Pearson's correlation test was used to assess the correlation of knowledge and attitude scores with other variables. Statistical significance was set at *p* < 0.05.

## 3 Results

Of the 263 distributed questionnaires, 207 were completed and returned (final response rate: 78.70%). Of the 207 participants who returned the questionnaires, 84 were general dentists, 50 were orthodontic residents, and 73 were senior dental students. Of all, 96 participants were males (46.4%) and 111 were females (53.6%). The demographic information of the respondents is presented in Table 2.

**Table 2:** Demographic information of the respondents.

Variable	Number	Percentage
Gender		
Male	96	46.4
Female	111	53.6
Dental students	73	35.3
Orthodontic residents	50	24.2
General dentists	84	40.6
University type		
Public	47	22.7
Private	76	36.7
Not a student	84	40.6
Professional practice		
Private clinic	67	32.4
Public clinic	46	22.2
Private office	25	12.1
Don't work	69	33.3
Professional clinical work experience		
1–3 years	106	51.2
3–5 years	12	5.8
More than 5 years	20	9.7
Not working	69	33.3

### 3.1 Knowledge

**Figure 1:**
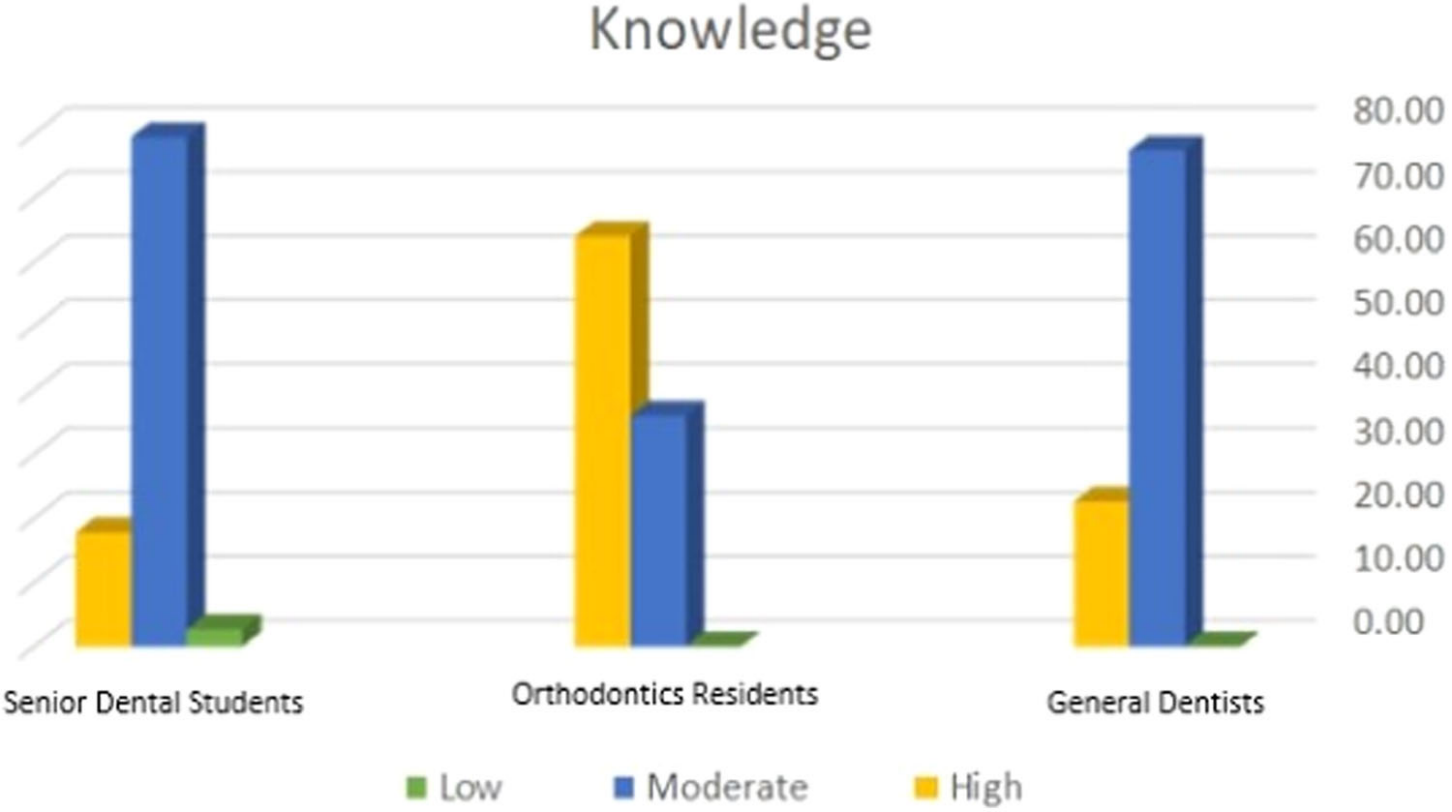
Frequency distribution of the knowledge scores of general dentists, senior dental students, and orthodontic residents about OSA.

The mean knowledge score of all participants was 15.99 (range 1–23). Table 3 compares the knowledge level of the participants according to different variables. Figure 1 demonstrates the frequency distribution of the participants on the basis of their knowledge level about OSA.

**Table 3:** Knowledge scores of the participants.

Variable	Mean	Type of test	*p* value
Dental students	15.20	First one‐way ANOVA and then post hoc Bonferroni	*p* value of one‐way ANOVA < 0.001
Orthodontic residents	18.28		
General dentists	15.32		
University type			
Public	1.82	Independent *t*‐test	0.680
Private	16.84		
Gender			
Female	15.85	Significance test of Pearson's correlation coefficient	0.001
Male	16.11		
Work experience			
No experience	92.87	First Kruskal−Wallis test and then the Mann−Whitney *U* test for pairwise comparisons	*p* value of Kruskal−Wallis = 0.002
1–3 years	118.74		
3–5 years	84.67		
More than 5 years	75.90		
Practice			
Private clinics	136.98	First Kruskal−Wallis test and then the Mann−Whitney *U* test for pairwise comparisons	*p* value of Kruskal−Wallis < 0.001
Public clinics	122		
Private office	133.70		
Not working	53.78		

The mean knowledge scores of general dentists and dental students were significantly lower than those of orthodontic residents (*p* < 0.001). However, there was no significant difference in the mean knowledge score of senior dental students and general dentists (*p* = 1).

The mean knowledge score was significantly higher in dentists with 1–3 years of professional clinical experience compared to those with over 5 years of clinical experience (*p* = 0.02), but no significant difference was found between other groups (*p* > 0.05).

Furthermore, the knowledge level was the highest among dentists who worked in private clinics, followed by private offices and public clinics.

Females had a significantly higher knowledge score than males (*p* < 0.001). There was no significant difference in knowledge score based on age (*p* = 0.578), type of university (*p* = 0.680), or time of graduation (*p* = 0.21).

### 3.2 Attitude

**Figure 2:**
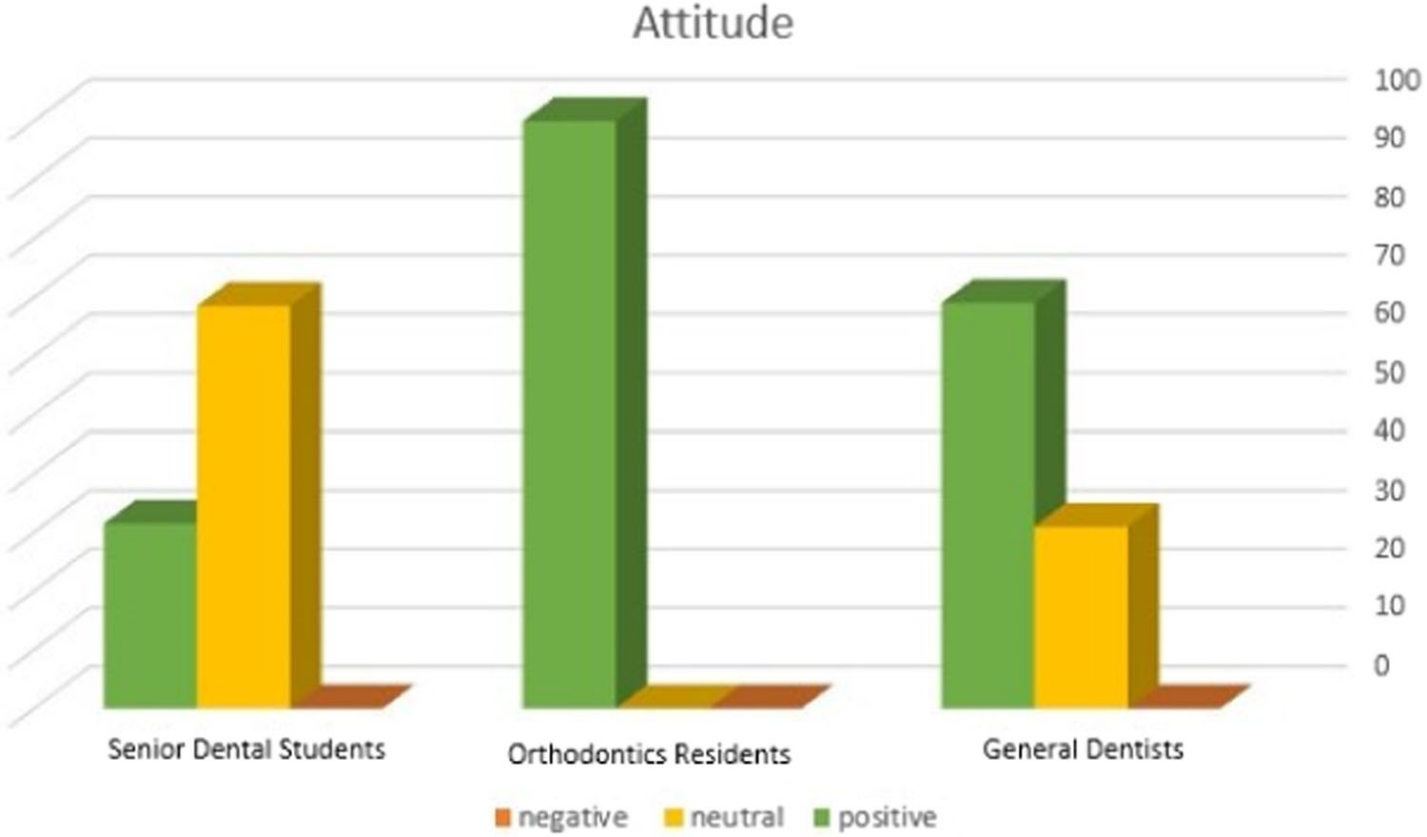
Frequency distribution of the attitude scores of general dentists, senior dental students, and orthodontic residents toward OSA.

Figure 2 illustrates the frequency distribution of the participants according to their attitude score toward OSA, and Table 4 displays the variations in the attitude scores of different groups. The mean attitude score was the highest in orthodontic residents followed by general dentists and dental students (*p* < 0.001).

**Table 4:** Attitude scores of the participants.

Variable	Mean	Type of test	*p* value
Dental students	23.05	First one way ANOVA and then Post hoc Bonferroni	*p* value of one‐way ANOVA < 0.001
Orthodontic resident	28.36		
General dentists	25.80		
University type			
Public	24.95	Independent *t*‐test	0.712
Private	25.36		
Gender			
Female	25.27	Significance test of Pearson's correlation coefficient	0.376
Male	25.61		
Work experience			
No experience	53.78	First Kruskal−Wallis test and then the Mann−Whitney *U* test for pairwise comparisons	*p* value of Kruskal−Wallis < 0.001
1–3 years	126.31		
3–5 years	152.33		
More than 5 years	130.05		
Practice			
Private clinics	139.07	First Kruskal−Wallis test and then the Mann−Whitney *U* test for pairwise comparisons	*p* value of Kruskal−Wallis < 0.001
Public clinics	97.0		
Private office	88.16		
Not working	92.87		

Attitude had no significant correlation with the type of attending university (*p* = 0.712), age (*p* = 0.409), or time of graduation (*p* = 0.21).

Clinicians working in private clinics had a higher attitude score than those working in public clinics (*p* < 0.001). Additionally, individuals with 1–3 years of work experience had a significantly higher level of attitude compared to those without any experience (*p* < 0.05); no significant difference was found between other groups (*p* > 0.05).

No significant difference was found between males and females regarding the attitude score (*p* = 0.376).

## 4 Discussion

OSA is an underdiagnosed dilemma, and its prevalence is often underestimated (Fuhrman et al. 2012; Lorenzi‐Filho, Genta, and Drager 2017). Tarasiuk et al. claimed that 2%–4% of the adult population is affected by OSA. However, health providers can detect only 10% of such patients (Kim and Kim 2020b). Failure to diagnose and treat OSA can bring about serious health consequences. Early diagnosis and orthodontic treatment in children may decrease the need for surgery in adulthood.

The lower level of knowledge and attitude of general dentists and dental students compared with orthodontic residents may be because OSA is not included in the undergraduate dental educational curriculum. Although the knowledge of dentists about OSA in different countries has been the topic of several investigations (Kale, Kakodkar, and Shetiya 2020; Amara Swapna et al. 2019; Vuorjoki‐Ranta et al. 2016; Alharbi et al. 2021; Simmons et al. 2021; Keramida, Kotakidou, and Kouratzi 2016), only one study was found in this regard in Iran (Shafiei et al. 2020).

Controversy exists regarding the knowledge and attitude of students toward OSA. A literature review indicated good knowledge but a lack of clinical experience of dentists regarding OSA (Nguyen 2023). Additionally, Swapna et al. indicated a lack of knowledge of senior dental students and general dentists in Riyadh, Saudi Arabia, about OSA (Amara Swapna et al. 2019). Kale et al. also assessed the dentists' knowledge, attitude, and practice in India. Their results displayed that despite the poor knowledge level and practice of dentists, they had a favorable attitude toward OSA (Kale, Kakodkar, and Shetiya 2020).

In contrast to the present findings, Shafiei et al. reported poor knowledge but a positive attitude among Iranian dental students and specialists toward OSA (Shafiei et al. 2020). This discrepancy may be because they examined only one dental faculty in Iran (Shahid Beheshti Medical University); whereas Iranian dental faculties nationwide were addressed in the present study.

Some previous studies suggested a positive correlation between the clinicians' age and years of professional experience with a higher level of knowledge and attitude (Nguyen 2023; Keramida, Kotakidou, and Kouratzi 2016); however, the present study found no such relationship. Instead, this study revealed that dentists with 1–3 years of work experience had a significantly higher knowledge score than those who had been working for over 5 years, but there was no significant difference among other groups; this may be because dentists with 1–3 years of work experience still have a fresh memory as not much time has passed since their graduation, compared to those with more than 5 years of experience. Since dentists do not receive any postgraduation training regarding OSA in Iran, their knowledge in this respect probably fades over time.

Additionally, individuals with 1–3 years of work experience had a significantly better attitude toward OSA compared to those without any experience, although there was no significant difference among other groups.

As a limitation, comparison of the present results with previous investigations was not possible as there were no similar studies on the knowledge and attitude of general dentists, orthodontic residents, and dental students toward OSA in Iran.

## 5 Conclusion

The present findings indicated that general dentists had insufficient knowledge about OSA, which could result in under‐diagnosis or misdiagnosis of patients with OSA. This finding highlights the need for additional education and training for dentists to properly identify and refer such patients to orthodontists or sleep specialists. Future research is recommended to include a larger sample size from all dental faculties of Iran.

## Author Contributions

Data collection, data analysis and interpretation, drafting the manuscript, submission: Mina Shekarian. Developing and designing the study, revising the manuscript: Masood Feizbakhsh. Developing and designing the study, revising the manuscript: Mehdi Rafie.

## Conflicts of Interest

The authors declare no conflicts of interest.

## Supporting information

Supporting information.

## Data Availability

If anyone requires the data from our research, kindly email the first author at shekarianmina@gmail.com or mina.shekarian@dnt.mui.ac.ir. We will promptly provide the requested data.
