# Supplementary material for: Knowledge and Attitude of General Dentists, Senior Dental Students, and Orthodontic Residents Toward Obstructive Sleep Apnea
Source: Clin Exp Dent Res. 2024 Sep 18;10(5):e931. doi: 10.1002/cre2.931 (PMC11411143; doi:10.1002/cre2.931)
Supplement: Supplementary file 1 — Supporting information. [file CRE2-10-e931-s001.docx]

**Questionnaire**

| Q1 | Do you know that sleep apnea means “complete or partial obstruction of the upper airway during sleep”?  Yes□ No□ I don’t know□ |
| --- | --- |
| Q2 | In your opinion, can dentists identify high-risk OSA patients using extra-oral radiographs?  Yes□ No□ I don’t know□ |
| Q3 | Can children also suffer from OSA?  Yes□ No□ I don’t know□ |
| Q4 | Do you think the common risk of OSA is lower in normal weight individuals?  Yes□ No□ I don’t know□ |
| Q5 | Do you think sleeping posture can be associated with OSA?  Yes□ No□ I don’t know□ |
| Q6 | OSA is more common among?  Males□ Females□ I don’t know□ |
| Q7 | Do you think that female hormones may prevent the development of OSA?  Yes□ No□ I don’t know□ |
| Q8 | Can alcohol consumption worsen OSA?  Yes□ No□ I don’t know□ |
| Q9 | Do you think craniofacial structures play a role in development of OSA?  Yes□ No□ I don’t know□ |
| Q10 | Can a short and wide neck serve as a risk factor for OSA?  Yes□ No□ I don’t know□ |
| Q11 | Do you think that size of the tongue can be associated with OSA?  Yes□ No□ I don’t know□ |
| Q12 | Is OSA more common in the elderly?  Yes□ No□ I don’t know□ |
| Q13 | Can daytime sleepiness be a symptom of OSA?  Yes□ No□ I don’t know□ |
| Q14 | Do patients with OSA have more bruxism during sleep than others?  Yes□ No□ I don’t know□ |
| Q15 | Can snoring be associated with OSA?  Yes□ No□ I don’t know□ |
| Q16 | Can night sweats be associated with OSA?  Yes□ No□ I don’t know□ |
| Q17 | Is periodic limb movement disorder a possible cause of OSA?  Yes□ No□ I don’t know□ |
| Q18 | Are morning headaches common in OSA patients?  Yes□ No□ I don’t know□ |
| Q19 | Can OSA lead to cognitive impairment?  Yes□ No□ I don’t know□ |
| Q20 | Can OSA aggravate cardiovascular problems?  Yes□ No□ I don’t know□ |
| Q21 | Can OSA cause systemic hypertension?  Yes□ No□ I don’t know□ |
| Q22 | Can diabetes mellitus be associated with OSA?  Yes□ No□ I don’t know□ |
| Q23 | Is untreated OSA associated with an increased risk of mortality?  Yes□ No□ I don’t know□ |
| Q24 | For some patients with OSA, an oral appliance is an effective treatment.  Strongly agree□ Agree□ I don't know□ Disagree□ Strongly disagree□ |
| Q25 | Dentists and physicians must work together for management of OSA.  Strongly agree□ Agree□ I don't know□ Disagree□ Strongly disagree□ |
| Q26 | Although OSA is a medical condition, dentists can identify, diagnose, and treat it.  Strongly agree□ Agree□ I don't know□ Disagree□ Strongly disagree□ |
| Q27 | Dentists can diagnose OSA by asking patients private questions.  Strongly agree□ Agree□ I don't know□ Disagree□ Strongly disagree□ |
| Q28 | Dentists can play a role in treatment of OSA.  Strongly agree□ Agree□ I don't know□ Disagree□ Strongly disagree□ |
| Q29 | OSA treatment with oral appliances can be performed by general dentists.  Strongly agree□ Agree□ I don't know□ Disagree□ Strongly disagree□ |

OSA: Obstructive sleep apnea
